# Effects of three permeases on arginine utilization in *Saccharomyces cerevisiae*

**DOI:** 10.1038/srep20910

**Published:** 2016-02-11

**Authors:** Peng Zhang, Guocheng Du, Huijun Zou, Jian Chen, Guangfa Xie, Zhongping Shi, Jingwen Zhou

**Affiliations:** 1 Key Laboratory of Industrial Biotechnology, Ministry of Education and School of Biotechnology, Jiangnan University, 1800 Lihu Road, Wuxi, Jiangsu 214122, China; 2 Synergetic Innovation Center of Food Safety and Nutrition, 1800 Lihu Road, Wuxi, Jiangsu 214122, China; 3 Zhejiang Guyuelongshan Shaoxing Wine Company, 13 Yangjiang Road, Shaoxing, Zhejiang, China

## Abstract

Arginine plays an important role in cellular function and metabolism. Arginine uptake mainly occurs through three amino acid permeases, Alp1p, Gap1p and Can1p, which act as both transporters and receptors for amino acid utilization. In this study, seven mutants were constructed with different combinations of permease deficiencies that inhibit arginine utilization. Their effects on arginine metabolism were measured. The three amino acid permeases were also individually overexpressed in wild-type (WT), Δ*alp1*Δ*gap1*Δ*can1* and Δ*npr1* strains. The growth and arginine utilization of Δ*can1*, Δ*gap1*Δ*can1* and Δ*alp1*Δ*gap1*Δ*can1* mutants were suppressed in YNB medium when arginine was the sole nitrogen source. Meanwhile, overexpression of Alp1p and Can1p enhanced growth and arginine utilization in WT, Δ*alp1*Δ*gap1*Δ*can1* and Δ*npr1*. Besides, overexpression of Can1p caused a 26.7% increase in OD_600_ and 29.3% increase in arginine utilization compared to that of Alp1p in Δ*alp1*Δ*gap1*Δ*can1*. Transcription analysis showed that the effects of three amino acid permeases on the arginine utilization and the regulation of related genes, were tightly related to their individual characteristics. However, their overall effects were different for different combinations of mutants. The results presented here suggest some possible synergistic effects of different amino acid permeases on regulation of amino acid utilization and metabolism.

Arginine plays an important role in cellular function and metabolism^1^. Arginine metabolism is modulated by the activities of various transporters that move arginine and its metabolites across the plasma and mitochondrial membranes^2^. In *Saccharomyces cerevisiae*, arginine is transported mainly by three amino acid permeases, *i.e.*, Can1p, Gap1p and Alp1p^3^. The specific arginine permease Can1p was the first one to be cloned and characterized^4^. Overexpression of Can1p can increase the efficiency of arginine uptake^5^. Gap1p is a general amino acid permease that can transport all naturally occurring L-amino acids, some D-amino acids, γ-aminobutyric acid^6^ and polyamines (putrescine and spermidine)^7^. Alp1p is considered to be a putative arginine permease^8^. Overexpression of Alp1p could also increase the efficiency of arginine uptake^9^.

In *S. cerevisiae*, arginine is first transported into the cell, then transported to the vacuole by vacuolar basic amino acid transporter 2 (Vba2p)^10^. Arginine biosynthesis genes, such as *ARG1, ARG3, ARG4, ARG5,6* and *ARG8*, are negatively regulated by arginine uptake, while arginine utilization genes such as *CAR1, CAR2* are positively regulated^11^. In addition to the direct induction of arginine metabolic gene expression caused by an influx of arginine, arginine uptake is also regulated by the general control of amino acid biosynthesis (GAAC)^12^. In a nitrogen-poor environment, arginine permease genes are activated by nitrogen catabolic repression (NCR) regulators Gln3p and Gat1p^13^. However, the mechanisms enabling cells to properly coordinate cellular arginine pools are not well understood^3^. Some of the previous works showed that GATA regulators were regulated by both NCR and target of rapamycin complex 1 (TORC1) dependent pathways^14^.

In response to different nutrient conditions, the TORC1 complex plays a key role in regulating cell growth and nutrient uptake^15^. It is a general regulator involved in cellular transcription, translation and induction of cell autophagy in yeast^16^. The regulation of nitrogen utilization in yeast by TORC1 complex occurs mainly through its roles in cell endocytosis by a negative phosphate kinase Npr1p, the arrestin-related protein Art1p, and the ubiquitin ligase Rsp5p^16^,^17^,^18^. In nitrogen-poor environments, the TORC1 pathway is blocked. Then some of the amino acid permeases are phosphorylated by Npr1p, activating the transporter protein function. When glutamine and other preferred nitrogen source are present, the TORC1 pathway is activated while the Npr1p phosphorylation is inhibited, arginine permeases could be polyubiquitined by ubiquitin ligase Rsp5p and its associated binding adaptor proteins. The permease is subsequently transported to the vacuole for degradation^19^.

The mechanisms of regulation for amino acid permeases have been well analyzed^3^,^19^. However, for amino acid permeases with the ability to transport the same amino acid, knowledge of the precise regulation scheme is essential for further investigation. In this study, different arginine-related amino acid permease disruption mutants were constructed to investigate the roles of the three arginine permeases in arginine utilization, metabolism and transcriptional regulation. The three amino acid permeases were overexpressed in wild type (WT), Δ*alp1*Δ*gap1*Δ*can1* and Δ*npr1 S. cerevisiae* strains. Under culture conditions with arginine as the sole nitrogen source, the transcription of the three arginine permeases, arginine metabolism and amino acid permease regulation genes were analyzed. The results showed the effects of three amino acid permeases on the arginine utilization and the regulation of related genes, were tightly related to their individual characteristics. The results presented here suggested that arginine permeases disruption combinations might have a role in the precise regulation of arginine utilization and metabolism by changing arginine uptake patterns.

## Results

### Arginine utilization in different arginine permease disrupted strains

**Figure 1:**
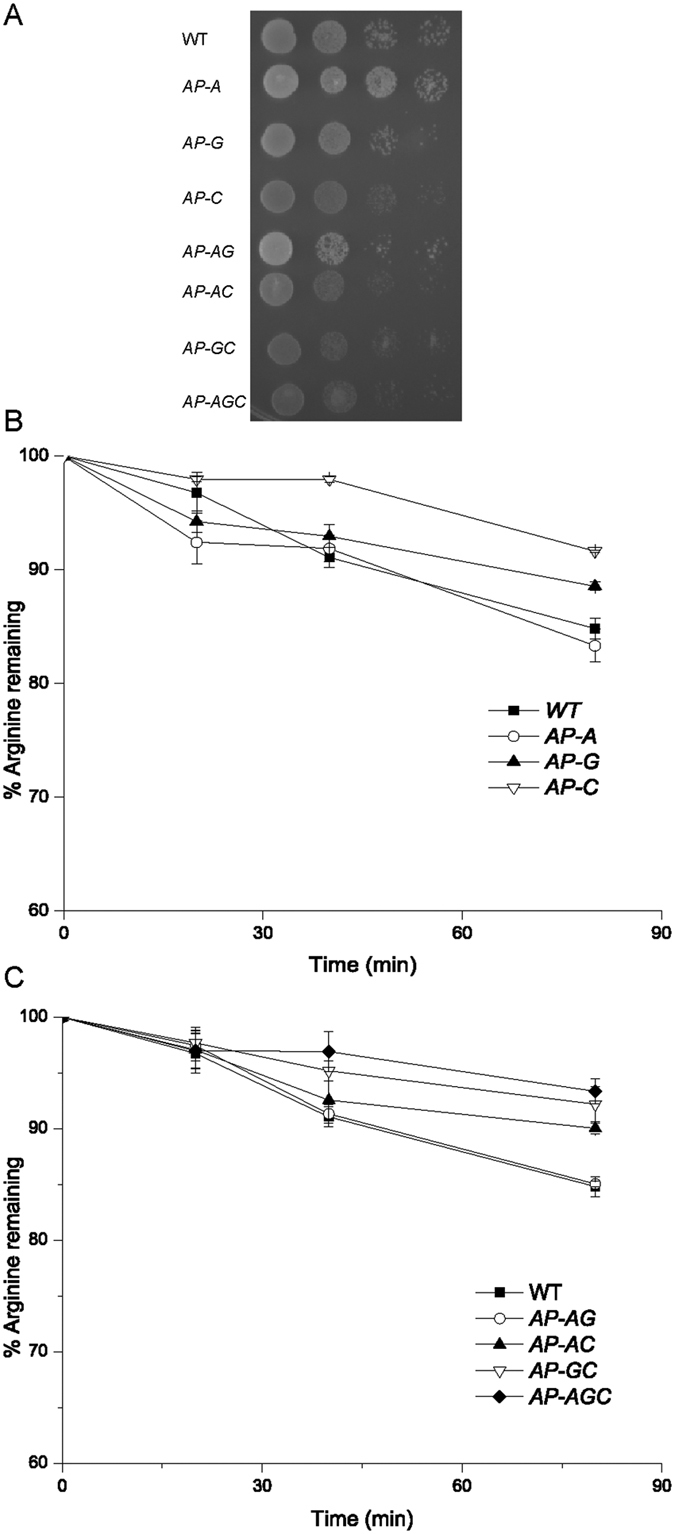
Effects of knockout of three arginine permeases on arginine utilization. Eight strains were cultured in YNB + ammonium sulfate medium to log phase. The cells were used for the following assays: (**A**) Spotting assay of different arginine permeases disruption mutants. Cells were cultured on solid plates containing YNB + 2 mM arginine and cultured for 48 h. (**B**) Arginine remaining of the individual disruption mutants of three amino acid permeases. Cells collected and transferred to YNB + 2 mM arginine medium for arginine remaining test. (**C**) Arginine remaining of double or triple disruption mutants of three amino acid permeases. Cells collected and transferred to YNB + 2 mM arginine medium for arginine remaining test. The WT strain was used as a control. Error bars represent standard deviations (*n* = 3).

Seven mutants were used to investigate the influence of different arginine permease disruptions on arginine utilization. The results showed that the growth of these mutants with *CAN1* disruption was more suppressed than the WT strain. The triple disruption strain *AP-AGC* (Δ*alp1*Δ*gap1*Δ*can1*) could still survive in YNB (yeast nitrogen base without amino acids and ammonia) medium with 2 mM arginine as the sole nitrogen source (Fig. 1A). Besides, arginine was present in the culture broth of individual permease disrupted mutants (Fig. 1B) and double/triple permease disrupted mutants (Fig. 1C) at 80 min. The WT strain, *AP-A* (Δ*alp1*), *AP-AG* (Δ*alp1*Δ*gap1*) showed more than 15.2% consumption of external arginine, while *AP-G* (Δ*gap1*), *AP-AC* (Δ*alp1*Δ*can1*) showed a 10.0% to 12.0% reduction. Arginine utilization by *AP-C* (Δ*can1*), *AP-GC* (Δ*gap1*Δ*can1*) and *AP-AGC* were severely suppressed (only 7.2% to 8.0% consumption) at 80 min.

### Effects of permease disruption on transcription of arginine metabolism-related genes

**Figure 2:**
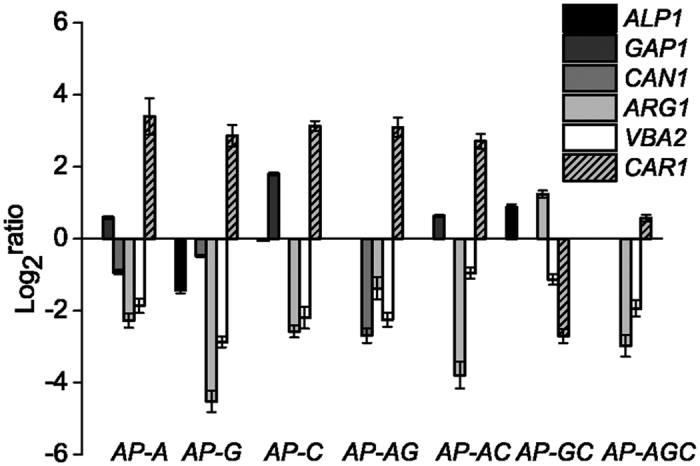
Transcriptional analysis of arginine permeases and metabolic genes. The mutants were cultured in YNB + ammonium sulfate medium to log phase. Cells were then collected and transferred to YNB + 10 mM arginine medium for 2 h. The expression levels of the three amino acid permeases, arginine metabolism and amino acid permease regulation genes were measured. The WT strain was used as a control. Data were normalized to the *ACT1* gene. Error bars represent standard deviations (*n* = 3).

Arginine uptake is the first step in arginine metabolism. To address the transcriptional influence of the different arginine permeases disruption on arginine metabolism, arginine permeases and arginine metabolism related genes were assayed by qRT-PCR (Fig. 2). The expression level of the *CAN1* was down-regulated in *AP-A* (1.9-fold), *AP-G* (1.4-fold) and *AP-AG* (6.5-fold) mutants. The expression level of *GAP1* was up-regulated in the *AP-A* (1.5-fold), *AP-C* (3.5-fold) and *AP-AC* (1.6-fold) mutants. The expression level of *ALP1* was down-regulated in the *AP-G* (2.7-fold), *AP-AC* (0.1-fold) and *AP-GC* (1.2-fold) mutants. The expression levels of *ARG1* and *VBA2* were down-regulated at least 2.6-fold in all mutants except for the *ARG1* was up-regulated in *AP-GC* (2.36-fold). However, the *CAR1* was up-regulated at least 1.5-fold in these mutants except for *AP-GC* (6.5-fold down-regulated).

### Effects of arginine metabolism gene disruption on the transcription of permeases

**Figure 3:**
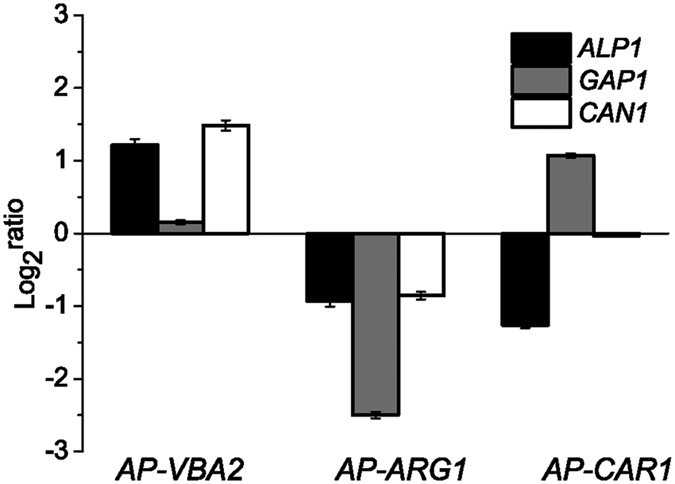
Transcriptional analysis of permeases in arginine metabolism-related genes disruption mutants. The mutants were cultured in YNB + ammonium sulfate medium to log phase. Cells were then collected and transferred to YNB + 10 mM arginine medium for 2 h. The expression levels of three amino acid permease genes were measured in *AP-VBA2, AP-ARG1* and *AP-CAR1*. The WT strain was used as a control. Data were normalized to the *ACT1* gene. Error bars represent standard deviations (*n* = 3).

In order to analyze interactions between arginine metabolism genes and arginine permeases genes, the expression level of the three arginine permeases were measured in *AP-ARG1* (Δ*arg1*), *AP-CAR1* (Δ*car1*) and *AP-VBA2* (Δ*vba2*) (Fig. 3). Compared to the WT strain, the expression level of *ALP1* was up-regulated in *AP-VBA2* (2.3-fold), while down-regulated in *AP-ARG1* (1.9-fold) and *AP-CAR1* (2.1-fold). The expression level of *GAP1* was up-regulated in *AP-VBA2* (1.1-fold) and *AP-CAR1* (2.1-fold), whereas it was down-regulated by 5.6-fold in *AP-ARG1.* The expression level of *CAN1* was up-regulated in *AP-VBA2* (2.8-fold), while down-regulated in *AP-ARG1* (1.8-fold) and *AP-CAR1* (1.0-fold).

### Effects of permease overexpression on arginine utilization and cell growth

**Figure 4:**
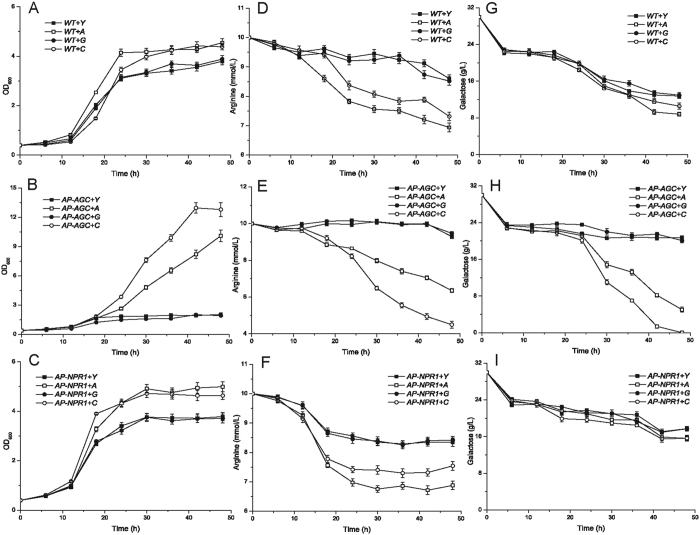
The effects on arginine utilization of overexpressing three arginine permeases. The overexpression strains were cultured in YNB + ammonium sulfate medium to log phase. Cells were then diluted to OD_600_ = 0.3~0.5, and cultured in YNB + 10 mM arginine with 30 g/L galactose as the carbon source for 48 h. *WT *+* Y, AP-AGC *+* Y* and *AP-NPR1* + *Y* were used as controls, respectively. Error bars represent standard deviations (*n* = 3). (**A–C**) The OD_600_ values while overexpressing three arginine permeases in WT, *AP-AGC* and *AP-NPR1*, respectively. (**D–F**) Extracellular arginine utilization while overexpressing three arginine permeases in WT, *AP-AGC* and *AP-NPR1*, respectively. (**G–I**) Galactose consumption while overexpressing three arginine permeases in WT, *AP-AGC* and *AP-NPR1*, respectively.

To further determine the effects of the three amino acid permeases on arginine utilization and cell growth, they were overexpressed in WT, *AP-AGC* and *AP-NPR1*. The cell growth, concentrations of arginine and galactose, were measured after 48 h (Fig. 4). Compared to the control strain, overexpression of Alp1p and Can1p caused an increase in OD_600_ in WT, *AP-AGC* and *AP-NPR1*. Overexpression of Can1p alone caused a 26.7% increase in OD_600_ compared to Alp1p in *AP-AGC* at 48 h. Arginine utilization was more efficient by overexpression of Alp1p in WT and *AP-AGC*. However, overexpression of Can1p increase the arginine utilization by 29.3% compared to that of Alp1p in *AP-AGC* at 48 h.

### Effects of permease overexpression on the transcription of arginine metabolism-related genes

**Figure 5:**
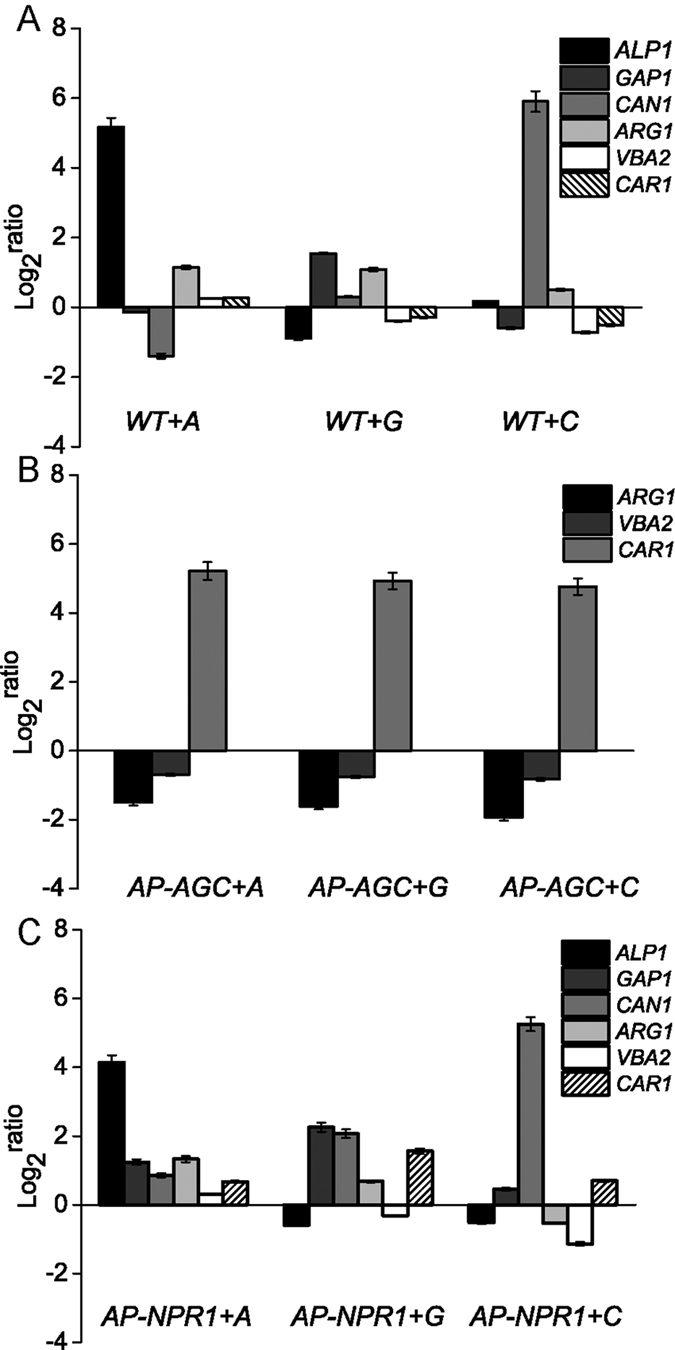
Effects of overexpressing three arginine permeases on the transcriptional levels of arginine permeases and metabolism-related genes. Overexpression strains were cultured in YNB + ammonium sulfate medium to log phase. Cells were then collected and transferred to YNB + 10 mM arginine medium with 30 g/L galactose as the carbon source for 2 h. *WT *+* Y, AP-AGC* + *Y* and *AP-NPR1* + *Y* were used as controls, respectively. Data were normalized to the *ACT1* gene. Error bars represent standard deviations (*n* = 3). (**A**) Gene expression while overexpressing three amino acid permeases in WT strain. (**B**) Gene expression while overexpressing three amino acid permeases in *AP-AGC*. (**C**) Gene expression while overexpressing three amino acid permeases in *AP-NPR1*.

In order to find out the reasons for the influence of overexpression of the three amino acid permeases on arginine utilization and cell growth, the expression levels of the three amino acid permeases, arginine metabolism and arginine permease regulation genes were measured in WT, *AP-AGC* and *AP-NPR1* (Fig. 5). Compared to the control strains, overexpression of one arginine permease gene showed different influence on the other two arginine permeases. The expression level of *ALP1* gene was down-regulated in *WT *+* G* (1.9-fold). The expression level of *GAP1* gene was up-regulated in *AP-NPR1 *+* A* (2.4-fold). The expression level of *CAN1* gene was down-regulated in *WT *+* A* (1.8-fold), while up-regulated in *AP-NPR1 *+* A* (2.6-fold) and *AP-NPR1 *+* G* (4.2-fold). Furthermore, the rest of arginine permease genes showed no significant expression level changes (all up-/down-regulations were below 1.5-fold).

Overexpression of three arginine permeases also showed different influence on arginine metabolism related genes. The expression level of *ARG1* gene was up-regulated at least 1.6-fold while *ALP1* and *CAN1* genes were individually overexpressed in WT and *AP-NPR1*. However, the *ARG1* gene was down-regulated at least 2.8-fold while three arginine permease genes were overexpressed in *AP-AGC*. The expression level of *VBA2* gene was down-regulated while *CAN1* was overexpressed in WT (1.7-fold) and *AP-NPR1* (2.2-fold), and down-regulated at least 1.6-fold while three arginine permeases genes were overexpressed in *AP-AGC*. The expression level of *CAR1* was up-regulated at least 1.6-fold and 30.4-fold while three arginine permeases were overexpressed in *AP-NPR1* and *AP-AGC*, respectively. The rest of arginine metabolism genes showed no significant expression level changes (up/down-regulated by less than 1.5-fold).

## Discussion

Amino acids are transported via different amino acid permeases^3^. However, permeases that transport the same amino acid may display different specific roles in amino acid transport^20^. The results present here demonstrated that different combinatorial disruption and/or overexpression of three arginine-related permeases showed different roles in promoting cell growth, arginine utilization and the transcription of arginine permeases and arginine metabolic genes. The results revealed that the characteristics and combined effects of three amino acid permeases on the regulation of arginine metabolism. It also provided useful clues for the regulation of amino acids metabolism through rational combinational engineering of different amino acid permeases.

In *S. cerevisiae*, arginine is transported into cells mainly through three different amino acid permeases^3^. However, how those permeases contribute synergistically to arginine uptake and their combinatorial effects remained unclear. Previous works on NH_4_^+^ and L-phenylalanine permeases with different combinations of permease disruptions provided a comprehensive knowledge of the combined and individual roles of these permeases^20^,^21^. It was also reported that genotype Δ*gap1*Δ*can1* could substantially eliminate arginine uptake^9^. Here, a systematic investigation on the three arginine permeases revealed combinatorial effects in both arginine utilization and its transcriptional regulation (Figs 1,2). The combinatorial effects of three arginine permeases could be dependent on the individual characteristics of three amino acid permeases and their corresponding upstream regulation processes.

These three amino acid permeases are not only used as transporters for amino acids, but also as receptors. Gap1p is reported to be an amino acid transport receptor^22^. Can1p is known as a GAAC- and NCR-regulated permease^3^,^23^. Since Alp1p and Can1p are considered to be transporters of cationic amino acids^24^, the fact that the expression level of *GAP1* was up-regulated in the *AP*-*AC* mutant and down-regulated in the *WT *+* A* and *WT *+* C* strains may indicate an antagonistic effect while *ALP1* and *CAN1* were disrupted or overexpressed. It also showed that disruption of *ALP1* or *CAN1* accompanied with *GAP1* disruption showed a synergetic effect on each other. Furthermore, overexpression of Alp1p supported adequate arginine uptake ability as Can1p (Fig. 4D–F), suggested the limited effect of *ALP1* disruption on arginine utilization might be the result of lower promoter activity^9^.

TORC1 regulates ubiquitin-mediated endocytosis via Npr1p-mediated phosphoinhibition of a ubiquitin ligase adaptor^16^. Since Gap1p and Can1p are two of the most important Npr1-regulated amino acid permeases^16^,^25^. Disruption of *NPR1* affects the expression level of arginine metabolic genes, while the expression level of these three arginine metabolism related genes were consistent in *AP-AGC*. This means that Npr1p influences the arginine transport function and regulation via arginine permeases. Previous reports showed that Alp1p was an effective transporter for arginine uptake^9^. Here, we further demonstrated that Alp1p are highly efficient for arginine utilization. However, Can1p showed higher capacity for arginine transport. Although the Alp1p was highly homologous to Can1p^26^, their characteristics and regulation mechanisms might be different, thus lead to different regulation patterns of arginine transport and metabolism.

In summary, this study demonstrated the combinatorial disruption and overexpression effects of the three arginine-related amino acid permeases on arginine utilization and metabolism. Although the amounts of amino acid permeases have been identified and characterized^3^, clearer differences between amino acid permeases that can transport the same amino acid still need to be characterized. This study provided systematic insight on fine regulation of arginine metabolism by the characteristics, combinatorial effects and upstream regulation processes of three arginine permeases. The results also showed that all of these amino acids permeases were well orchestrated and tightly linked between each other in the precise regulation of arginine uptake and metabolism process.

## Materials and Methods

### Strains, media and culture conditions

*S. cerevisiae* strains used in this study are listed in Table 1. *S. cerevisiae* BY4741 was used as the initial strain^27^. Strains were first cultured in YNB + ammonium sulfate medium (1.6 g/L yeast nitrogen base without amino acids or ammonium, 20 g/L glucose and 5 g/L ammonium sulfate); appropriate supplements (25 mg/L leucine, 25 mg/L histidine, 25 mg/L methionine and 25 mg/L uracil) were added when required. For analysis of arginine utilization, cells were cultured in YNB + arginine media in which arginine was the sole nitrogen source. YPD + G418 plates (10 g/L yeast extract, 20 g/L peptone, 20 g/L glucose, 20 g/L agar, 200 mg/L G418 sulfate) were used for selection of G418 resistance transformants.

For arginine utilization analysis, strains were cultured in YNB + ammonium sulfate medium at 30 °C until OD_600_ = 0.6 ~ 1.0. Cells were then harvested, washed twice with YNB medium without ammonium or amino acids, and the solutions were adjusted to OD_600_ = 1.0 and transferred to a YNB + 2 mM arginine medium^19^. Arginine was monitored for 80 min. For overexpression of the three amino acid permeases, cells were first cultured in YNB + ammonium sulfate medium to the log phase, then they were diluted to OD_600_ = 0.3~0.5 (to reduce the lag phase) in YNB + 10 mM arginine with 30 g/L galactose as the sole carbon source, The concentration of arginine and OD_600_ were monitored for 48 h.

### Plasmid construction and gene disruptions

The pYES2 plasmid was purchased from Invitrogen (Carlsbad, CA). Three arginine permease genes *ALP1, GAP1* and *CAN1* were amplified by PCR and cloned into pYES2 using the primer pairs *ALP1-EX-F*/*ALP1-EX-R, GAP1-EX-F*/*GAP1-EX-R* and *CAN1-EX-F*/*CAN1-EX-R*, respectively. The upstream and downstream homologous regions of the three amino acid permease genes, *ALP1, GAP1, CAN1* and *VBA2* were PCR-amplified using primer pairs *ALP1*-P1/*ALP1*-P2 and *ALP1*-P3/*ALP1*-P4, *GAP1*-P1/*GAP1*-P2 and *GAP1*-P3/*GAP1*-P4, *CAN1*-P1/*CAN1*-P2 and *CAN1*-P3/*CAN1*-P4, *VBA2-*P1*/VBA2-*P2 and *VBA2-*P3*/VBA2*-P4, *ARG1-*P1*/ARG1-*P2 and *ARG1*-P3*/ARG1*-P4, *CAR1-*P1*/CAR1-*P2 and *CAR1*-P3*/CAR1*-P4 and *NPR1-*P1*/NPR1-*P2 and *NPR1-*P3*/NPR1-*P4, respectively. Then these PCR products and four *loxP* marker cassette plasmids, pUG6 (*kan*^*r*^), pUG27 (*his5* + ) and pUG73 (*LEU2*), pUG72 (*URA3*), were used to construct the disruption cassette using a fusion PCR method^28^,^29^. The disruption cassettes were confirmed by Sanger sequencing and then transformed into cells using the LiAc method^30^. Disruption strains were confirmed by amplification using flanking primer pairs *ALP1*-VF/*ALP1*-VR, *GAP1*-VF/*GAP1*-VR, *CAN1*-VF/*CAN1*-VR, *VBA2*-VF/*VBA2*-VR, *ARG1*-VF/*ARG1*-VR, *CAR1*-VF/*CAR1*-VR and *NPR1-VF/NPR1-VR,* respectively. All of the primers are listed in Table 2.

### Spotting assay

Yeast cells were cultured in the YNB + ammonium sulfate medium at 30 °C to log phase, then serially diluted and spotted on YNB + 2 mM arginine. The plates cultured at 30 °C for 48 h. Individual spotting assays were performed in triplicates (at once). Each set of triplicates were repeated for twice^31^.

### Amino acid analysis

Analyses of arginine were performed using the Agilent HPLC system 1260 (Palo Alto, CA) equipped with an ODS-2 Hypersil column (4.6 × 150 mm × 5 μm) (Thermo Scientific, CA) as previously described^32^.

### RNA preparation and DNA synthesis

Yeast cells were cultured in the YNB + ammonium sulfate medium at 30 °C to log phase, transferred to YNB medium with 10 mM arginine and 30 g/L galactose or 20 g/L glucose as the sole carbon source for 2 h. Cells were washed twice with double-distilled water and stored at −80°C until RNA preparation. The procedures for RNA extraction and cDNA synthesis have been described previously^33^.

### Real-time quantitative PCR (qRT-PCR) assay

Primers used for qRT-PCR were designed by Beacon Designer 7.0 (Table 2). qRT-PCR experiments were performed using SYBR^®^ Premix Ex *Taq*^TM^ kit (Takara, Dalian, China). Parameters for PCR were: pre-incubation at 95 °C for 30 s; 40 cycles of amplification at 95 °C for 5 s, 55 °C for 20 s; finally, cooling at 50 °C for 30 s. Reactions were conducted using a LightCycler 480 II Real-time PCR instrument (Roche Applied Science, Mannheim, Germany) and were run in triplicate. Mean values were used for further calculations. The fold change was determined by the 2^−ΔΔC^_T_ method normalized to the *ACT1* gene^34^.

## Additional Information

**How to cite this article**: Zhang, P. *et al*. Effects of three permeases on arginine utilization in *Saccharomyces cerevisiae. Sci. Rep.* **6**, 20910; doi: 10.1038/srep20910 (2016).

## Figures and Tables

**Table 1:** Strains of *Saccharomyces cerevisiae* used in this work.

Name	Parent	Genotype
WT	*BY4741*	*MATa Δhis3-1 Δleu2 Δmet15 Δura3*
*AP-A*	*BY4741*	*MATa Δhis3-1 Δleu2 Δmet15 Δura3 ALP1::LEU2*
*AP-G*	*BY4741*	*MATa Δhis3-1 Δleu2 Δmet15 Δura3 GAP1:: kanMX*
*AP-C*	*BY4741*	*MATa Δhis3-1 Δleu2 Δmet15 Δura3 CAN1:: kanMX*
*AP-AG*	*BY4741*	*MATa Δhis3-1 Δleu2 Δmet15 Δura3 GAP1:: HIS5 ALP1::LEU2*
*AP-AC*	*BY4741*	*MATa Δhis3-1 Δleu2 Δmet15 Δura3 ALP1::LEU2 CAN1:: kanMX*
*AP-GC*	*BY4741*	*MATa Δhis3-1 Δleu2 Δmet15 Δura3 GAP1:: kanMX CAN1::HIS5*
*AP-AGC*	*BY4741*	*MATa Δhis3-1 Δleu2 Δmet15 Δura3 GAP1:: HIS5 ALP1::LEU2 CAN1:: kanMX*
*AP-VBA2*	*BY4741*	*MATa Δhis3-1 Δleu2 Δmet15 Δura3 VBA2::URA3*
*AP-ARG1*	*BY4741*	*MATa Δhis3-1 Δleu2 Δmet15 Δura3 ARG1:: kanMX*
*AP-CAR1*	*BY4741*	*MATa Δhis3-1 Δleu2 Δmet15 Δura3 CAR1:: kanMX*
*AP-NPR1*	*BY4741*	*MATa Δhis3-1 Δleu2 Δmet15 Δura3 NPR1:: kanMX*
*WT* + *Y*	*BY4741*	*MATa Δhis3-1 Δleu2 Δmet15 Δura3 pYES2*
*WT* + *A*	*BY4741*	*MATa Δhis3-1 Δleu2 Δmet15 Δura3 pYES2-ALP1*
*WT* + *G*	*BY4741*	*MATa Δhis3-1 Δleu2 Δmet15 Δura3 pYES2-GAP1*
*WT* + *C*	*BY4741*	*MATa Δhis3-1 Δleu2 Δmet15 Δura3 pYES2-CAN1*
*AP-AGC* + *Y*	*BY4741*	*MATa Δhis3-1 Δleu2 Δmet15 Δura3 GAP1:: HIS5 ALP1::LEU2 CAN1:: kanMX pYES2*
*AP-AGC* + *A*	*BY4741*	*MATa Δhis3-1 Δleu2 Δmet15 Δura3 GAP1:: HIS5 ALP1::LEU2 CAN1:: kanMX pYES2-ALP1*
*AP-AGC* + *G*	*BY4741*	*MATa Δhis3-1 Δleu2 Δmet15 Δura3 GAP1:: HIS5 ALP1::LEU2 CAN1:: kanMX pYES2-GAP1*
*AP-AGC* + *C*	*BY4741*	*MATa Δhis3-1 Δleu2 Δmet15 Δura3 GAP1:: HIS5 ALP1::LEU2 CAN1:: kanMX pYES2-CAN1*
AP-NPR1 + Y	*BY4741*	*MATa Δhis3-1 Δleu2 Δmet15 Δura3 NPR1:: kanMX pYES2*
AP-NPR1 + A	*BY4741*	*MATa Δhis3-1 Δleu2 Δmet15 Δura3 NPR1:: kanMX pYES2-ALP1*
AP-NPR1 + G	*BY4741*	*MATa Δhis3-1 Δleu2 Δmet15 Δura3 NPR1:: kanMX pYES2-GAP1*
AP-NPR1 + C	*BY4741*	*MATa Δhis3-1 Δleu2 Δmet15 Δura3 NPR1:: kanMX pYES2-CAN1*

**Table 2:** Oligonucleotides used for quantitative RT-PCR and gene disruption.

Gene	Name	Sequence (5′-3′)
**Oligonucleotides for qRT-PCR**		
*ALP1*	*ALP1*-F	CGAGGATGATGCTGCTAAGG
	*ALP1*-R	CAAGTCCAAGTCTGTCAAGTC
*GAP1*	*GAP1*-F	GAAGCACCACTTGAAGAATAG
	*GAP1*-R	CCAGAGCCATAACCATAGC
*CAN1*	*CAN1*-F	GTGATGAAGATGAAGGAGAAG
	*CAN1*-R	TGCGTGACAGAATATGCC
*ARG1*	*ARG1*-F	TTCTTACGAGGCAGGTATCTTG
	*ARG1*-R	AAGTCTTGTTGTCGGTGTAGG
*CAR1*	*CAR1*-F	ACGGAATTAGAGCCCTCAATG
	*CAR1*-R	GGAATCTGTTCGCCTGGAC
*VBA2*	*VBA2*-F	CGGTAGGCAGTCTCATAACAG
	*VBA2*-R	TAGACCAATCAACCAGTTCGG
*ACT1*	*ACT1*-F	TTATTGATAACGGTTCTGGTATG
	*ACT1*-R	CCTTGGTGTCTTGGTCTAC
**Oligonucleotides for genes disruption**		
*ALP1*	*ALP1*-P1	TGTACCAAAAGTTGCACAAAAAT
	*ALP1*-P2	GCGTACGAAGCTTCAGCTGGGCAATAATCGCAACACTACAAT
	*ALP1*-P3	CAGATCCACTAGTGGCCTATGCCCAAATCCTTGCTCAACTCT
	*ALP1*-P4	CCTCGCCTCAGTAGATGTAG
*GAP1*	*GAP1*-P1	GCCTCACTAATCTACCCATTG
	*GAP1*-P2	GCGTACGAAGCTTCAGCTGGCTGTCCTTGGTCTGTTCTT
	*GAP1*-P3	CAGATCCACTAGTGGCCTATGCCGAACAACGACAAAAAAAGAC
	*GAP1*-P4	GAATCCCAACAATTATCTCAACAT
*CAN1*	*CAN1*-P1	TCTTGTCCCTTATTAGCCTTGAT
	*CAN1*-P2	GCGTACGAAGCTTCAGCTGTTGTTTTTACAGGAGTTAAGAAGT
	*CAN1*-P3	CAGATCCACTAGTGGCCTATGCCCCTCATAAGTCATACACCGAA
	*CAN1*-P4	TCAACTATAAATCCGAGGGCTAC
*VBA2*	*VBA2*-P1	GGGGTTTCACATATTGTTCTAT
	*VBA2*-P2	GCGTACGAAGCTTCAGCTGTGATTAAATTCGTTACCAATCGTT
	*VBA2*-P3	CAGATCCACTAGTGGCCTATGCTATATACCTATGCCCTTGACC
	*VBA2*-P4	GATTCCCTATATGTGCGGTA
*CAR1*	*CAR1*-P1	GGTAAAGCTACGCATACTGTCT
	*CAR1*-P2	GCGTACGAAGCTTCAGCTGCTTGATAGTAGTTATTGTTATTGATAT
	*CAR1*-P3	CAGATCCACTAGTGGCCTATGCCCTTTTATCAAAATAAGCATTCTCT
	*CAR1*-P4	GGTTATTCCGATCTTGTTCCT
*ARG1*	*ARG1*-P1	ACTAACACAATTAAATAATCGCCAT
	*ARG1*-P2	GCGTACGAAGCTTCAGCTGTATTTTATGCAATTATGTGTATTTC
	*ARG1*-P3	CAGATCCACTAGTGGCCTATGCGTCCGCTAGTTCATCGCCTC
	*ARG1*-P4	GGTACTTGCCGTTTAGCTTT
*NPR1*	*NPR1*-P1	TTTTCGTTATCAATGACGCATC
	*NPR1*-P2	GCGTACGAAGCTTCAGCTGAATGTTTCGTAGAGCTTTCCT
	*NPR1*-P3	CAGATCCACTAGTGGCCTATGCATAAGCGTCCCCACTTTTATT
	*NPR1*-P4	TTTTGCTGCCTGTGACATTATGGA
**Oligonucleotides for genes disruption verification**		
*ALP1*	*ALP1*-VF	CTATGTTGTTGCCCACCGAT
	*ALP1*-VR	GGATACGGGAACATCTCCACTT
*GAP1*	*GAP1*-VF	CTGCTCACTAGAATCGTAATC
	*GAP1*-VR	TGTTGGCTGTTCAATCTCC
*CAN1*	*CAN1*-VF	CTATTCGGAGATACAGGCAAC
	*CAN1*-VR	ACCCGTTTGTAAATAGAATCAGC
*VBA2*	*VBA2*-VF	TCCGACCAGTAACCTTGTG
	*VBA2*-VR	TTCTGCTTACTACTATTAACCTT
*CAR1*	*CAR1*-VF	ACCAAACCGTGTAGGCAA
	*CAR1*-VR	TTGGGATTTACACGACAACGAC
*ARG1*	*ARG1*-VF	CCATTATACACGCTATTATCGTT
	*ARG1*-VR	TGCTTCTGCGTAATATCGTCCT
*NPR1*	*NPR1*-VF	TAAACGGAATAGTCGCGCATT
	*NPR1*-VR	AGTCAATGGCTTTTATACCCTT
**Oligonucleotides for overexpression of three arginine permeases**		
*GAP1*	*GAP1*-EX-F	GCGAGCTCTAAAAAATGAGTAATACTTCTTCGT
	*GAP1*-EX-R	CGGGATCCCATTAACACCAGAAATTCCAG
*CAN1*	*CAN1*-EX-F	GGGGTACCGGCATAGCAATGACAAATTC
	*CAN1*-EX-R	GCTCTAGACTATGCTACAACATTCCAAAAT
*ALP1*	*ALP1*-EX-F	GGGGTACCATGGATGAAACTGTGAACATAC
	*ALP1*-EX-R	CGGGATCCATTATGAAAGGACATCCCAAACT

*Underlined letters represent the overlap of *loxP* plasmids and primers.
